# Optimal combinations of acute phase proteins for detecting infectious disease in pigs

**DOI:** 10.1186/1297-9716-42-50

**Published:** 2011-03-17

**Authors:** Peter MH Heegaard, Anders Stockmarr, Matilde Piñeiro, Rakel Carpintero, Fermin Lampreave, Fiona M Campbell, P David Eckersall, Mathilda JM Toussaint, Erik Gruys, Nanna Skall Sorensen

**Affiliations:** 1 Innate Immunology Group, National Veterinary Institute, Technical University of Denmark, 1790 Copenhagen V, Denmark; 2 Department of Molecular and Cellular Biochemistry and Biology, Faculty of Science, University of Zaragoza, Zaragoza, Spain; 3 Institute of Infection, Immunity and Inflammation, College of Medicine, Veterinary Medicine & Life Science, University of Glasgow, Glasgow G61 1QH, UK; 4 Department of Pathobiology, Faculty of Veterinary Medicine, Utrecht University, Utrecht, The Netherlands; 5 Current Address: Division of Immunology and Allergy, Clinical Immunology Unit, University Hospital, Geneva, Switzerland; 6 Current Address: pigCHAMP Pro Europa S.A., Gremios Segovianos 13, Pol Ind Hontoria, 40195 Hontoria, Segovia, Spain; 7 Current Address: University of Aberdeen, Rowett Institute of Nutrition and Health, Aberdeen, Scotland, UK

## Abstract

The acute phase protein (APP) response is an early systemic sign of disease, detected as substantial changes in APP serum concentrations and most disease states involving inflammatory reactions give rise to APP responses. To obtain a detailed picture of the general utility of porcine APPs to detect any disease with an inflammatory component seven porcine APPs were analysed in serum sampled at regular intervals in six different experimental challenge groups of pigs, including three bacterial (*Actinobacillus pleuropneumoniae, Streptococcus suis, Mycoplasma hyosynoviae*), one parasitic (*Toxoplasma gondii*) and one viral (porcine respiratory and reproductive syndrome virus) infection and one aseptic inflammation. Immunochemical analyses of seven APPs, four positive (C-reactive protein (CRP), haptoglobin (Hp), pig major acute phase protein (pigMAP) and serum amyloid A (SAA)) and three negative (albumin, transthyretin, and apolipoprotein A1 (apoA1)) were performed in the more than 400 serum samples constituting the serum panel. This was followed by advanced statistical treatment of the data using a multi-step procedure which included defining cut-off values and calculating detection probabilities for single APPs and for APP combinations. Combinations of APPs allowed the detection of disease more sensitively than any individual APP and the best three-protein combinations were CRP, apoA1, pigMAP and CRP, apoA1, Hp, respectively, closely followed by the two-protein combinations CRP, pigMAP and apoA1, pigMAP, respectively. For the practical use of such combinations, methodology is described for establishing individual APP threshold values, above which, for any APP in the combination, ongoing infection/inflammation is indicated.

## Introduction

The acute phase protein (APP) response is an innate reaction towards tissue injury and follows rapidly (6-12 h) after onset of any disease compromising tissue homeostasis, for example infections, trauma, inflammation with various etiologies and some tumors. It can also be induced by injection of microbial molecules (peptidoglycan, lipopolysaccharide) and pro-inflammatory cytokines [1-3]. The APP response involves substantial changes in the serum concentrations of numerous proteins, mainly as a result of changes in their hepatic synthesis rates, although other organs and tissues also show local APP responses [4]. The APPs are typically present in the μg/mL to mg/mL range in serum and plasma from an affected individual.

The APP response is thus a robust indicator of disease and easily measurable in a blood sample. It has been used as a very useful diagnostic tool for many years especially in human medicine [5-7] but also increasingly in veterinary medicine [8], especially for companion and sports animals [9,10]. We have characterized the APP response in pigs undergoing experimental infections [11-15] and the correlation between APP concentrations and disease in pigs in herds of different health status has also been studied [16-22]. It is well established that some APPs react to a lesser extent than others to the same infection/inflammatory stimulus [8] reflecting different induction sensitivities of different APPs. Conversely, APPs may also react differently to different types of stimuli as reported for SAA (serum amyloid A), Hp (haptoglobin) and alpha-1-acid glycoprotein in cattle, which were all found to be more sensitive indicators of acute than of chronic inflammation [23,24]. Other examples of this include porcine Hp reflecting more closely the extent of lung damage in respiratory diseases than CRP (C-reactive protein) [25], pigMAP (pig major acute phase protein) that was reported not to react to infection with PRRSV (porcine respiratory and reproductive syndrome virus) in naturally infected pigs [18], and increased Hp being associated with lesions due to enzootic pneumonia caused by *Mycoplasma hyosynoviae *but not with lesions due to pleuritis (caused by *Actinobacillus pleuropneumoniae*) at slaughter [16]. The use of more than one APP to increase the sensitivity of disease detection was indicated in some of these studies [18,25] and generalized in the suggestion by Gruys et al. [26] of using both rapidly reacting as well as slowly reacting APPs for detection of disease with increased sensitivity.

Although much useful information has been gained from these studies, here we seek to answer the question of which combination of pig APPs can be used generally to give the most sensitive detection of ongoing disease in the pig. The aim of the study was therefore to define combinations of APPs that can be tested for use in real life monitoring of infections in pig herds where, typically neither the nature of infection nor the course of the infection is known. As explained above, the threshold for initiating an APP response varies between proteins and diseases, as does the speed at which an acute phase reaction resolves. In addition to this, the magnitude of the reaction is also dependent on the APP/disease combination. We therefore studied the acute phase response of a number of specific pig APPs to several relevant infectious agents as well as to aseptic inflammation in experimental groups of pigs. The choice of APPs was based on the following criteria: the acute phase changes of the APPs should be well described, substantial and reproducible, and reliable assays should be generally available for their measurement in serum. Furthermore both positive and negative APPs should be included.

The serum panel employed consisted of more than 400 samples, obtained from three bacterial, one viral, and one parasitic infection and one aseptic inflammation group, including infections with the prevalent pathogenic agents *Actinobacillus pleuropneumoniae *(*A.p.*), *Streptococcus suis *(*S. suis*)*, Toxoplasma gondii *(*T. gondii*)*, Mycoplasma hyosynoviae *(*M. hyos.*), porcine reproductive and respiratory syndrome virus (PRRSV) and as a model inflammation, pigs injected aseptically with turpentine.

Immunochemical analyses of seven different acute phase proteins, four positive (CRP, Hp, pigMAP and SAA) and three negative (albumin (Alb), transthyretin (TTR), and apoA1) were performed on all samples using the best available assays in four different European laboratories. Advanced statistical treatment of the data was performed in a two-step procedure, including defining cut-offs for a positive reaction and calculating detection probabilities for single APPs as well as for all possible APP combinations in order to select the combination of APPs that was most sensitive for detecting any of the infections/inflammation by strictly objective criteria.

By selecting APP's that complement each other during the progression of an infection, we aimed to construct a measure of infection that was more sensitive than any individual APP, over a wide period of the disease progression.

## Materials and methods

### Animal groups

Test serum samples were obtained from consecutive time points from a number of well-controlled and well-characterized experimental infection experiments as well as from a group of pigs undergoing induced aseptic inflammation. Serum samples were obtained prior to and from early, intermediate and late time points during infection/inflammation. Sampling and experimental groups were defined as follows: pigs should be more than one month of age, the whole infection period (pre-infection, during infection, post-infection) should be covered by the sampling, a statistically adequate number of samples/animals should be included for each infection, data on clinical signs and pathology should be available, and samples from subclinically infected and virus-infected pigs should be included.

All Danish pigs were from specific pathogen free (SPF) herds. Breeds were Danish Yorkshire/Danish Landrace (for the *S. suis *and *T. gondii *groups, see below) or crossbreds between Yorkshire/Landrace sows and Hampshire/Duroc boars (*A.p. *and *M. hyos. *groups) and the age was from 4/5 weeks and upwards (see below). Spanish pigs were used for the inflammation group and were crossbreds between Large White, Landrace and Pietrain pigs and 20 weeks of age. Before inoculation, pigs were acclimatized for at least 1 week in isolation units in groups of 3-6 animals with free access to water, and fed commercial feed without antibiotic growth promoters. All animal experiments were conducted in accordance with local legislation (Danish Animal Experiments Inspectorate and Ethical Committee for Animal Research at the University of Zaragoza) and were executed according to best practices and legislation with veterinary supervision to avoid unnecessary suffering.

*S. suis*: Five SPF pigs with no history of *Streptococcus suis *serotype 2 infection and approx. 6 weeks of age were infected by subcutaneous injection with a *S. suis *serotype 2, ribotype I isolate (strain SS02-0119)[27], 1 mL (10^10 ^CFU) in the back of the neck. Blood samples were obtained at days -8, 0, 1, 2, 5, 8, 12 and 14 after inoculation (pi). All pigs were euthanized and necropsied at day 14 pi.

*A.p.*: Twelve pigs, approx. 8 weeks of age, were inoculated (aerosol inhalation)[28] with an *A. p. *serotype 4 isolate. Blood samples were obtained at days 0, 3, 7, 10, 14 and 18 pi. When needed pigs were individually treated with antibiotics as described elsewhere [29]. Pigs were euthanized and necropsied at day 49 pi.

*T. gondii*: Five pigs, approx. 7 weeks of age, were inoculated by i.v. injection with 10^4 ^tachyzoites of *T. gondii *isolate SVS P14 [14]. Blood samples were obtained at days 0, 3, 6, 8, 10, 14, 17 and 21 pi. Autopsy was performed at day 27 pi.

PRRSV: Three pigs, 21 weeks of age, were inoculated intranasally with a European PRRSV isolate (DK-111/92)[30]. Blood samples were obtained at days -12(or -11), 0, 1, 2, 3, 4, 5, 7, 9, 11, 16 and 25 after inoculation.

*M. hyos.*: Nine pigs, 4½ weeks of age, were inoculated intranasally with 2 × 1.5 mL of 10^10 ^colour changing units/ml of a cloned *M. hyos. *field strain (Mp 927cl) [31] and blood samples from dpi -8, -2, 4, 6/7, 9, 12 and 14/15 were obtained.

Inflammation: Five pigs at 20 weeks of age were subjected to aseptic inflammation by s.c. injection of 0.3 mL of turpentine/Kg body weight distributed equally on each side of the neck [12] and blood samples were obtained at 0, 12 h, 24 h, 36 h, 48 h, 3 days, 4 days, 7 days, 10 days and 14 days pi.

### Blood samples

All blood samples were collected without anti-coagulant and allowed to clot (at room temperature for 2 hours or at 4°C overnight), before retrieval of serum by centrifugation. Serum samples were stored below -20°C until use.

### Acute phase protein assays

The serum panel was blind-tested for the concentrations of the positive APPs CRP, pigMAP, Hp and SAA and the negative APPs apoA1, TTR and albumin, using immunoassays.

Briefly, albumin, pigMAP and ApoA1 were determined by radial immunodiffusion [32] in 1% agarose gels containing specific rabbit polyclonal antisera and using a porcine serum as a secondary standard. The concentration of these proteins in the secondary standard was previously determined by radial immunodiffusion using the purified proteins as standard [12,33,34]. Intra- and inter-assay coefficients of variation were below 5%.

The concentration of CRP, TTR, SAA and Hp was measured by ELISA. Serum CRP was measured as described in Sorensen et al. [15]. Microtiter plates were coated with phosphoryl choline coupled BSA (BSA-CP) and blocked with milk-powder in saline [35]. Samples and standards were diluted in 50 mM Tris, 0.9% NaCl, 10 mM CaCl_2_, 0.1% Tween 20 (TBS-CT buffer), and bound CRP was detected using an in-house anti-pig CRP monoclonal antibody, followed by a peroxidase-labelled goat-anti-mouse antiserum (Jackson Immuno research Laboratories). All washings and additions of secondary reagents were done in TBS-CT buffer. The ELISA was developed using 50 mM citric acid, pH 4.0, 0.1 mM ABTS (2,2 Azino-bis(3-ethylbenzthiazoline-6-sulfonic acid)), 0.01% H_2_O_2 _as a color substrate and the absorbance read at 405 nm. For the measurement of TTR [11,15], microtiter plates were coated with serum samples and purified human TTR (standard)(Sigma-Aldrich, Poole, UK) and blocked with non-fat dried milk in assay buffer (0.12 M NaCl, 0.02 M Na2HPO4, 0.1% (v/v) Tween 20, pH 4.0). Bound TTR was detected with sheep anti-human TTR antiserum, followed by a peroxidase conjugated anti-sheep IgG (Sigma, Poole, UK). All washing and detection steps were performed using assay buffer. The ELISA was developed using TMB substrate solution and the absorbance was read at 450 nm. Porcine Hp was analysed by sandwich ELISA essentially as described before [15], using an in-house monoclonal antibody against porcine Hp as the catching antibody. A pool of pig serum calibrated against a porcine Hp standard from Saikin Kagaku Co. Ltd. (Japan) was used as in-plate standard. Samples were run in duplicate and the absorbance was read at 490 nm subtracting 650 nm. Finally, the concentration of SAA in the samples was assessed by a sandwich ELISA from Tridelta Ltd. (Tridelta Development Ltd, Bray, Co. Wicklow, Ireland) in accordance with the manufacturer's instructions.

**Statistical treatment of data **(Detailed information on this part of the work may be obtained from the authors)

The aim of the statistical treatment was to establish a measure of the ability of single APPs as well as any combination of APPs to detect ongoing infection/inflammation. To do this, *detection probabilities*, based on *cut-off values *calculated for each APP, were computed and evaluated for each of the five treatment groups separately (sections "*Univariate analysis for calculation of single APP detection probabilities*" and "*Multivariate analysis for calculation of combined APP detection probabilities*"), and for all of these weighted together in a *performance index *for ongoing unknown infection/inflammation (section "*A global *performance index *for unknown infection/inflammation*").

#### Univariate analysis for calculation of single APP detection probabilities

Pre-treatment APP concentrations as derived from the experimental data were used to estimate *cut-off values *for each APP, i.e. the maximum (minimum for ApoA1, albumin and TTR) value that the APP concentration is expected to attain within a standard, one-sided 95% confidence interval in an animal not undergoing an infection. This was done by approximating the observed pre-infection values with a normal distribution having (estimated) mean μ and variance σ^2 ^for each APP (except SAA, see below, Results). For a positive APP the *cut-off value *is then , where *c *is the 0.95 percentile in the standard normal distribution, and *n *is the number of animals in the data used to estimate μ and σ^2^. For a negative APP (i.e. ApoA1, albumin and TTR), the cut-off is . An animal is then classified as undergoing infection/inflammation if the concentration of a given positive APP measured in a sample from this animal is above the cut-off value for the APP in question (and below for a negative APP). This classification does not indicate anything about the nature or the time course of the infection/inflammation.

In principle, the distribution of pre-infection values for any given APP should not vary between the treatment groups. This, however proved not to be the case here, and therefore specific cut-off values had to be calculated for each treatment group.

Cut-off values allowed the calculation of *detection probabilities*. The detection probability is defined as the probability, based on APP data, of classifying an animal as undergoing infection/inflammation given that the animal is actually undergoing infection/inflammation as defined by the experimental conditions. Thus, the detection probability equals the sensitivity of the measurement(s) of the given APP in revealing ongoing infection/inflammation using the experimentally defined infection/inflammation status of the animal as the "gold standard". For an APP measurement at time t after the start of the infection/inflammation, having mean *μ_t _*and variance *σ_t_^2^*, the detection probability *ρ_t _*for a positive APP is simply defined as the integral of the corresponding normal density above the cut-off value, i.e.

For negative APPs the modification of this expression is obvious.

#### Multivariate analysis for calculation of combined APP detection probabilities

A combined measurement of more than one type of APP is expected to yield higher detection probabilities. To quantify these gains in sensitivity, *combined detection probabilities *needed to be calculated for all combinations of two or more APPs. Albumin and TTR were disregarded due to inappropriate data structures for these two APPs (see Results), and thus calculations were based on Hp, CRP, ApoA1 and pigMAP only, using multivariate analysis of pre-infection measurements of these four APPs (using minus the pre-infection measurements for the negative APP ApoA1). As these measurements showed a clear pig effect they were approximated with a multivariate normal distribution, in which variance-covariance parameters were allowed to vary freely. This interdependence of APP concentrations was remedied by establishing a theoretical transformation making the APP concentration statistically independent. This was done by arbitrarily sequencing the APPs as CRP first, then Hp, ApoA1 and pigMAP. Then the difference between the true mean and the conditional mean of any of the APP measurements, given concentration measurements for the remaining APPs in the sequence, was added. For one-dimensional normal variables *X *and *Y *with means μ_x_, μ_y_, variances σ_x_^2^, σ_y_^2 ^and correlation ρ, these operations correspond to subtracting (ρσ_x_/σ_y_)( Y- μ_y_) from *X*. For any subset of the four APPs, a similar technique was applied, simply by deleting the APPs not included in the subset from the sequence of APPs. This transformation rendered all co-variances between APP measurements zero while retaining the original mean, and allowed the application of standard techniques for independent stochastic variables. Then, c*ombined cut-off *values were calculated for each APP in a combination in order to keep the combined probability that any APP in the combination show a value above its combined cut-off value below 5% given that the animal is not undergoing infection/inflammation. Thus, with *Y_j _*denoting the *j*'th transformed APP measurement, which has (estimated) mean μ_*j *_and variance σ_*j*_^2^, the decision rule for classifying the animal as 'infected' on the basis of a subset *J *of the four APPs considered, will be that

where μ_j _+ cσ_j _is the combined cut-off value for APP *j*. If *J *is all four APPs, c is equal to 2.23. For J consisting of two or three APPs, *c *is equal to 1.95 and 2.12, respectively.

Finally, the *combined detection probabilities *were corrected for the fact that the independence-giving transformation was estimated through the estimates of variances and covariances from the multivariate normal approximation of data. The transformation thus deviates from the theoretical transformation, making the transformed APP concentration measurements only approximately stochastically independent. Also, there was no longer a fixed number of animals used to estimate the variance, as the different treatment groups had different numbers of animals. To deal with these two issues, 50 000 sets of 4 APP measurements for the same number of animals as in the experiment were simulated from the estimated multivariate distribution, and transformed as described above, using the transformation derived from the empirical variance-covariance of the simulated data. The value of *c *was then adjusted so that 5% of the simulations were classified as "infected". Based on the simulation study, we adjusted the *c *value for the AP4 data with a factor of 1.18 while Mycoplasma, *Strep.suis*, Toxoplasma and Inflammation did not require adjustment of the *c *value. Combined detection probabilities were then estimated for all time points in the study and for all combinations of the four APPs through simulation studies (10 000 replications per time point and set of APPs).

#### *A global *performance index *for unknown infection/inflammation*

In practice, it may not be known which, if any, infection/inflammation is present and at which stage. The detection probabilities for single APPs as well as for APP combinations computed above were all related to a specific infection/inflammation. To generalise this into an estimate of the overall/global detection probability of a given APP or APP combination, a global *performance index *was constructed, scoring the overall ability of any APP or APP combination to detect the five types of infection/inflammation that were considered in this study. This was based on the assumptions that each of the five infection/inflammation types were equally likely to be the one resulting in significantly changed concentrations of any of the four APPs, and that a sample from any time point in the study period had the same probability of representing an animal undergoing infection/inflammation. We considered the detection probabilities as a function of time from infection/inflammation, and extrapolated the function linearly between time points where data were available to the end of the study period (see Additional file 1, Figure S2; note that the end of the study period is defined by a point after the ultimate time point equalling half the distance between the ultimate and the penultimate time points, to put similar weights on all observations). At time 0 (time of infection/start of inflammation), the probability of incorrect detection was set to 0.05, to conform to the 95% confidence limit used for constructing detection probabilities. For any combination of APPs, the probability of detection at a uniformly random time point within the study period, the performance index *I_J _*for a subset *J *of the four APPs then takes the form , where the summation extends over the five treatment groups, where *f_J,i _*is the extrapolated detection probability function for the set of APPs *J*, for detecting infection/inflammation *i*, and where ℓ_*i *_is the length of the study period for infection/inflammation *i*.

## Results

### Disease development in the experimental groups

The inoculation strain was re-isolated from relevant tissue in all infected animals and macroscopic lesions typical of the infection in question were found in all animals upon necropsy, except one in the *S. suis *group, and one animal in the *A.p. *group. With the exception of the PRRSV group, general clinical signs such as fever and loss of appetite were observed in all groups as were specific clinical signs, in the relevant groups, such as lameness (*S. suis*, *M. hyos.*), and coughing and sneezing (*A.p.*). In short, clinical signs showed up early (within first 24 h after inoculation) in the *S. suis, A.p. *and inflammation groups and later in *M. hyos. *(most animals at days 9-12 pi) and *T. gondii *(day 6 pi) groups. In the PRRSV group all animals were asymptomatic even if they all presented with PRRSV viraemia at days 3-4 pi. Absence of clinical signs is typical of PRRSV-infected animals of this age (21 weeks). For ethical reasons most of the *A.p. *infected animals (11 of 12) were treated with antibiotics at 27 h.

As the study objective was to establish the best APP combination for indicating ongoing infection/inflammation, irrespectively of clinical signs, all animals were included, even if severity of both clinical signs and pathology varied between individual pigs. The specific inoculation agent was re-isolated from all animals.

### Acute phase protein response kinetics

Pre-challenge concentrations and the derived cut-off values for Hp differed between different experimental groups (see Table 1), while the within-group animal-to-animal variation was not bigger than for the other APPs. Very low pre-challenge concentrations of Hp were seen for the *A.p. *and the *M. hyos. *groups while the *T. gondii*, *S. suis *and inflammation groups showed much higher pre-challenge values, and, consequently, higher cut-off values. This indicated that pre-challenge conditions such as age of pigs, origin and sanitary/microbial and stress status of pigs and housing of pigs had a big effect on Hp concentration. For some reason, this effect was not as pronounced with the other APPs, although pre-challenge effects were also clearly detectable with CRP (Table 1). It can be noted that while the inflammation and *S. suis *groups showed affected pre-challenge concentrations for all proteins, the *A.p. *group showed a specific pre-challenge elevation of CRP and the *T. gondii *group showed a specific elevation of Hp.

**Table 1:** Cut-off values as calculated for each of the different experimental groups for four of the APPs investigated (CRP: μg/mL, Hp: mg/mL, pigMAP: mg/mL, ApoA1: mg/mL)

	*A.p.*	*T. gondii*	*M. hyos.*	*S. suis*	Inflammation
CRP	23.70	14.41	0.55	30.82	19.39

Haptoglobin	0.004	1.54	0.007	0.94	0.85

pigMAP	0.45	0.59	0.82	1.55	0.93

ApoA1	1.84	2.06	2.92	0.96	1.62

**Figure 1:**
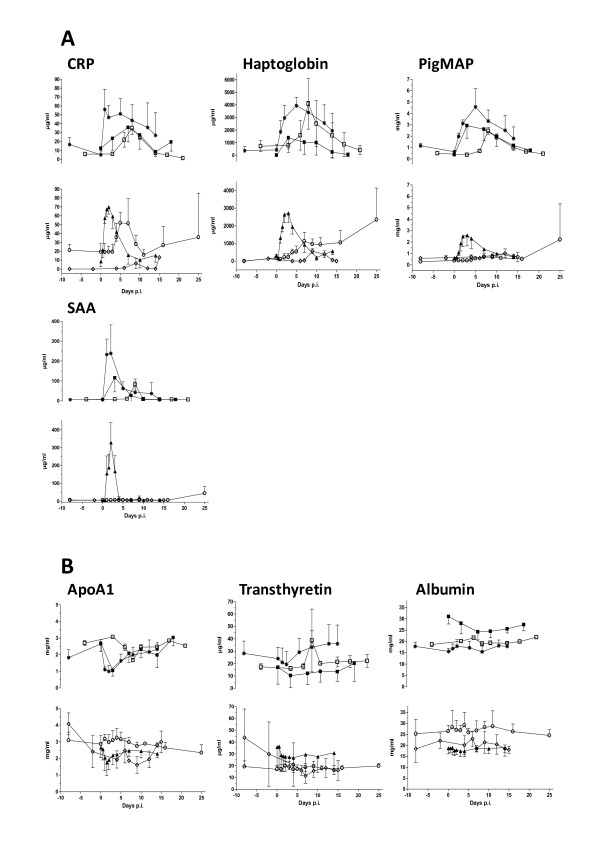
**Serum concentrations of the seven APPs from animals undergoing infection or inflammation as indicated, A: positive APPs, B: negative APPs**. Groups and group sizes: ■: *Actinobacillus pleuropneumoniae *(*n *= 12), ▲: Inflammation, (*n *= 5), ○: PRRS virus (*n *= 3), *: *Streptococcus suis *(*n *= 5), ◊: *Mycoplasma hyos. *(*n *= 9), □: *Toxoplasma gondii *(*n *= 5). Error bars indicate SD.

As seen in Figure 1a clear-cut APP responses for the positive acute phase proteins CRP, pigMAP, Hp and SAA were observed after infection with *A.p.*, *S. suis*, *T. gondii *as well as in response to inflammation, however infection with both PRRSV and *M. hyos*. generally induced very low responses with the notable exception of CRP which clearly increased after PRRSV infection. For the other groups responses reflected the development of clinical signs (see above), i.e. inflammation led to a very quick response, while responses were gradually slower in the order *S. suis*, *A.p. *and *T. gondii*. In addition, some variation between the responses of individual APPs was evident. This was most clearly seen with SAA which was an "all-or-none" responder with a large proportion of the samples not having SAA above the detection limit, and showing a very short-lived response. Also, there were subtle differences between the reactions of CRP, pigMAP and Hp to the different infections; for example CRP reacted more quickly in *S. suis *infected pigs than pigMAP and - as mentioned above - was the only APP induced by PRRSV. Also, Hp and CRP reacted more strongly to aseptic inflammation than did pigMAP, while Hp showed a lower response to *A.p. *than to *S. suis *and *T. gondii *compared to CRP and pigMAP which showed more similar responses to these three pathogens (see Figure 1a). For the negative APPs (Figure 1b), ApoA1 was the only protein showing a clear and transient decrease occurring rapidly for inflammation, *S. suis *and *A.p. *and later for *T. gondii*. The responses of negative APPs albumin and TTR were relatively weak with a large between-animal variation. TTR did show a weak transient decrease with *S. suis *and *M. hyos. *however was not affected by *T. gondii*, PRRSV and *A.p. *infection. During inflammation the serum concentration of TTR decreased rapidly and stayed depressed throughout the experiment. Albumin did appear to decrease in the course of *A.p. *infection, however did not react to any of the other infections.

Data from the PRRSV-infection experiment were excluded from further analyses due to the evident inability of this infection to induce any protein apart from CRP (see Figure 1) and therefore not contributing to defining the optimal APPs and APP combinations. The full set of data for individual animals is shown in Additional file 1, Figure S1.

### Detection probabilities for single APPs and APP combinations

Estimated detection probabilities for each infection/inflammation group are listed for CRP, Hp, pigMAP and ApoA1 and for all combinations of these in Table S1 (Additional file 1) and all single APP detection probabilities are shown in Figure S3a (Additional file 1). Albumin and TTR were not included due to their large between-animal variation and inconsistent responses, and SAA was also excluded, as calculation of a statistically meaningful cut-off value for this protein was not possible due to the pre-infection concentrations being below the detection limit of the assay and thus having zero variance.

**Figure 2:**
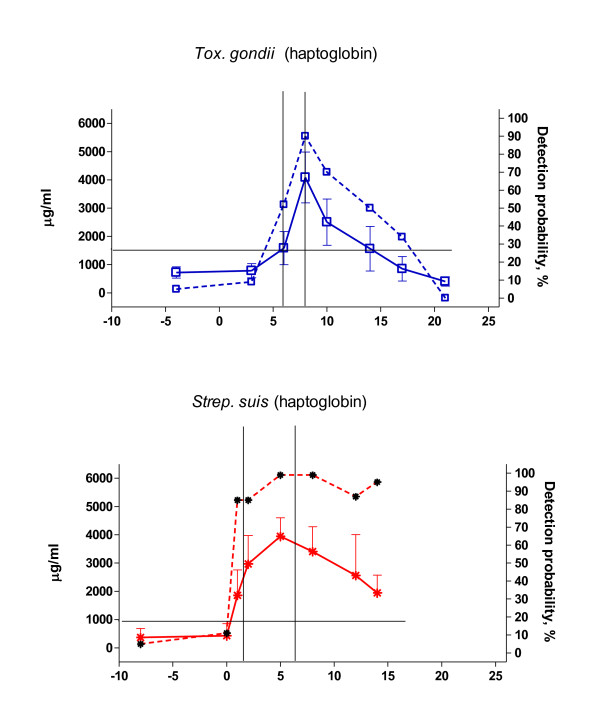
**Haptoglobin as induced by *Toxoplasma gondii *(upper) and *Streptococcus suis *(lower) infections depicted as haptoglobin concentrations (solid curve) and by haptoglobin detection probabilities (broken curve)**. Clinical phase (delimited by the vertical lines) is defined as the time period in which at least one animal in the group showed clinical signs. Horizontal line indicates the cut-off.

An example on the correlation between detection probabilities and actual APP concentrations with the clinical phase during infections is shown for haptoglobin in Figure 2 for the *S. suis *and *T. gondii *groups. As can be seen, the detection probabilities quite accurately reflect the much narrower clinical phase for the *T. gondii *infection as compared to the *S. suis *infection.

**Figure 3:**
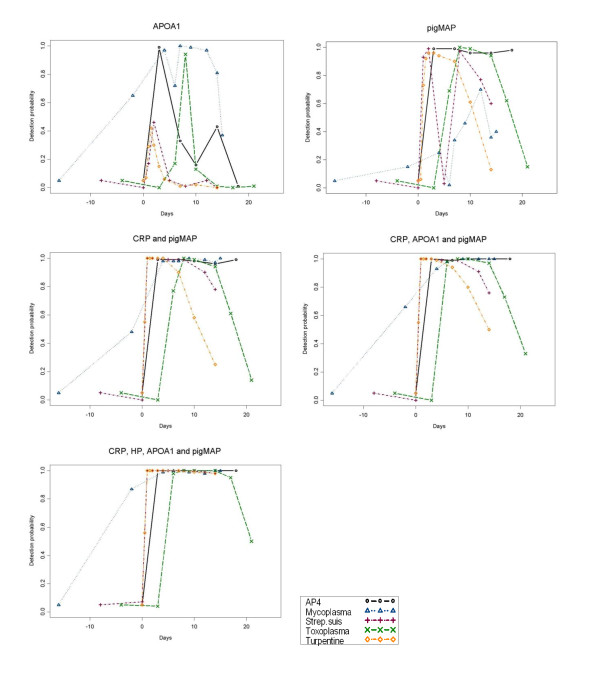
**Probability curves for worst (ApoA1) and best (pigMAP) overall performing single APP for all experimental groups combined, compared to best two-APP (CRP, pigMAP), three-APP (CRP, apoA1, pigMAP) and the one four-APP combination for each of the experimental groups**.

**Figure 4:**
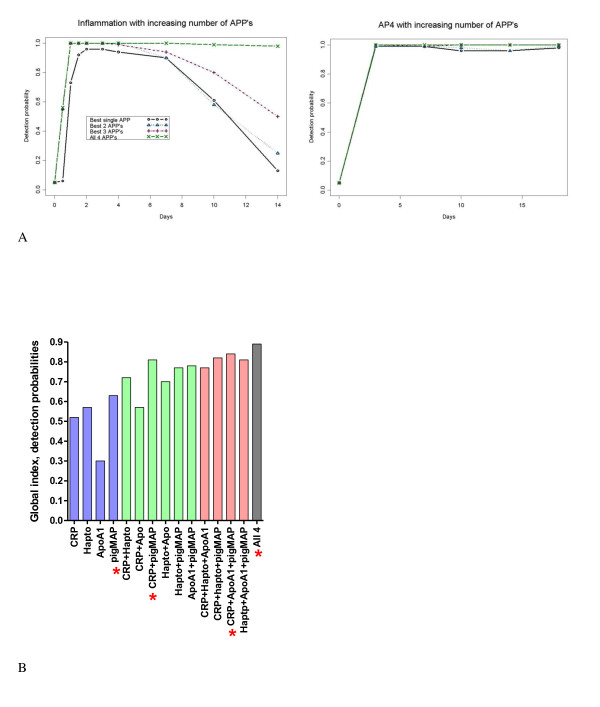
**Example of detection probabilities for best single and combined APPs with two experimental groups and global performance indexes for all single and  combined APPs**. A: Two examples of detection probability curves for best performing APP or APP combination for one protein, two proteins, three proteins and four proteins for two different experimental groups, inflammation (left), and *A.p. *(right). B: Bar graph showing global performance indexes for all single proteins and all multiple protein combinations, stars indicating the best performing APP or APP combination in each group (also see Additional file 1, Table S2).

Combined detection probabilities for APP combinations yielded a broader window of detection of all of the infection/inflammation groups (see Figure S3, Figure S4 and Table S1 (Additional file 1)). Figure 3 shows examples of detection probabilities going from one-dimensional to multivariate and showing the worst and the best one-protein APP, and the best two-, three- and the four-protein APP combination for all experimental groups. For *A.p.*, there was not much difference between using the best single APP and using any of the optimal APP combinations while, for the inflammation group there was always an effect of increasing the number of APPs and for the inflammation group there was always an effect of increasing the number of APPs (Figure 4a). Figure S3b (Additional file 1) shows that for *M. hyosyn.*, there was a big gain in going from one to two APPs but not much gain in increasing from two APPs to three or four, and for *S. suis*, there was an effect of using two APPs instead of one, but not much effect in increasing the number of APPs to three, although increasing it all the way to four APPs did have an effect. In the same figure it is seen that for *T. gondii*, there was no effect in increasing to two APPs, but there was effect of increasing the combination to three or four APPs. The full set of detection probability curves for all APP combinations is shown in Additional file 1, Figure S4.

### Overall performance of APPs and APP combinations (global performance index)

Estimated global performance indexes are shown in Table S2 (Additional file 1) and in Figure 4b. As the detection probability was fixed at 0.05 at time 0, the values in Table S2 (Additional file 1) should be compared with the upper bound of 0.935, which is due to the study design.

The average of the effect of increasing the number of APPs in the measurements was reflected in the increase in the global detection index for the best single, two-, three- and the four-APP combination, which were 0.63, 0.81, 0.84 and 0.89, respectively (graphically depicted in Figure 4b, also see Additional file 1, Table S2). This global performance of APPs and APP combinations was calculated by summing areas under the curve for detection probability curves for all infection/inflammation groups, for each APP and APP combination as described above. The best two-protein combination was CRP and pigMAP (0.81) closely followed by apoA1 and pigMAP (0.78), while the best three-protein combination was CRP, apoA1 and pig MAP (0.84), with the four-APP combination only slightly better (0.89). Thus, both of the best three-APP combinations and the four-APP combination were only marginally better than the CRP, pigMAP combination.

## Discussion

The data reported here give a wealth of information on the response of different APPs to different infections in the pig, complementing earlier studies in experimental models [11-15] and suggesting ways of using APPs for monitoring infections when type of the infection(s) as well as the infection starting points are unknown. The aim was to define the combination of APPs detecting any infection/inflammation with the highest possible sensitivity. The sensitivity of a given APP or APP combination for general detection of infection/inflammation will depend on the *generality *of the response of the APP(s) in question (the consistency of the response in a high proportion of animals exposed to a range of different - relevant - types of infections and inflammatory states), the *kinetics *of the response (the rapidity, peak time and extent of the response) and the *between-animal variation *(the extent to which the significance of the response is affected by variations in pre-infection levels and in response levels between individual pigs).

The approach taken here is general, not incorporating clinical data or taking biological differences between different infections into account, although they clearly give rise to different APP responses. The idea is that it would be beneficial if the APP measurement could also indicate subclinical infection (APPs have the potential to do just that, see for example Karreman et al. [36], Sorensen et al. [15] and Gerardi et al. [37]). It is also assumed that any combination of APP measurements giving a maximum detection probability as defined here, i.e. with no reference to occurrence of clinical signs and/or pathological changes, will also be the most globally sensitive combination for demonstrating any infection/inflammation, be it clinical or subclinical. In addition, the experiments included here are not comparable with respect to frequency and level of detail in recording clinical signs. From this it follows that data were treated under the assumption that all animals included in an experimental group were subject to the same course of infection/inflammation. Accordingly, the results of the calculations do not indicate to which extent the APPs can differentiate between individual animals being differently affected by the infection/inflammation (as e.g. indicated by differences in clinical responses). In other words, differences in reaction to the (same) stimulus by the individual pigs were incorporated into the calculations and accounted for the majority of the variations in the treatment groups.

The experimental groups covered a broad and relevant range of infections, and data on CRP, Hp, ApoA1 and pigMAP concentrations were included; Albumin and TTR concentrations showed a large animal-to-animal variation and negligible detection probabilities (not shown) and thus were excluded from further study, and for SAA a cut-off value could not be defined as its pre-infection serum concentration was below the detection limit of the assay (6 μg/mL). Data from the PRRSV group were excluded from analysis as only CRP showed any substantial response in this group.

The statistical treatment of data comprised a two-step procedure first defining cut-off values for the individual APPs and then deriving detection probabilities for single APPs and for combinations of APPs by multivariate analysis, both of these for each experimental group. As can be seen in Figure 3, the detection probability curves for the single worst (apoA1) and single best (pigMAP) performing APPs and for the best two-APP, three-APP and the single possible four-APP combination, clearly show that detection sensitivities for most challenge groups are much improved when increasing the number of APPs. To generalize this, a measure of overall (global) detection sensitivity for all of the experimental groups involved was constructed based on the summed area under the curve averaged over all of the infections in order to compare the *global performance indexes *for the different APP combinations. This measure gives the average probability of detecting, using the APP combination in question, any of the infections with all 5 infections equally probable.

This evaluation showed that APP combinations allowed the detection of disease more sensitively than any individual APP (best individual APP is pigMAP (0.63)). The global performance indexes for the best two-APP combination, the best three-APP combination and the four-APP combination were within a close range (0.81, 0.84 and 0.89, respectively) and close to the upper limit of the index (0.935). Indeed, it seems worthwhile to consider the two-protein combinations (especially CRP, pigMAP (0.81) and apoA1, pigMAP (0.78)), performing almost as well as the best three-protein combination. The benefit of choosing the best three-APP combination is that it includes both negative and positive APPs. The Hp, pigMAP combination had a similar global detection probability index (0.77), however it might be advisable to avoid Hp as its cut-off differed widely between the different treatment groups. Clearly, pre-challenge history (age of pigs, origin and sanitary/microbial status of pigs stress and housing of pigs) had a bigger effect on Hp than on the other APPs investigated. This confirms data reported on pig Hp in different pig herds [20], showing higher variability than CRP [16,17] and pigMAP [21]. Although cut-off values for CRP also varied considerably between experimental groups this was mostly due to one group having very low pre-challenge levels (*M. hyos.*).

While providing suggestions for which APPs to combine for sensitive detection of infection and inflammation in pigs, no generally applicable cut-off values, neither single-APP cut-off values nor combined cut-off values can be derived from this study. However, a method is provided for calculating combined cut-off values for each APP in an APP combination (see section "*Multivariate analysis for calculation of combined APP detection probabilities*"), based on pre-infection concentrations. Evidently, this favours the use of APPs that show little variation between animals (by increasing the detection probabilities for the APP in question) and APPs that show little variation between groups (or herds), by increasing the probability that a given cut-off value calculated from the pre-infection data from a collection of relevant samples is indeed applicable to the set of samples being evaluated.

The approach described here enables the use of the optimal APP combination for sensitive detection of infection/inflammation, by measuring each APP in the preferred combination and observing if any of the APPs are above (or below for negative APPs) their respective combination cut-off values. If specific circumstances make certain APPs more practical and/or advantageous to use than others, the methods presented here can also be used to calculate combination cut-off values for such a set of APPs.

Thus, a decision rule for defining an APP serum concentration as indicating infection/inflammation, irrespective of the type of infection or the time of disease progression was established, different APP combinations were evaluated and their optimally sensitive combinations were identified. While the methods are general, the results are dependent on the experimental structure that was used to obtain the APP data. Furthermore, the data were heterogeneous in the sense that it was not possible to establish a pre-infection distribution independent of infection type for each APP. Thus, for general, practical use, the definition of cut-off values, based on relevant pre-infection data is pivotal, necessitating that a relevant group of non-infected animals are available and that herd/management effects can be accounted for, as such effects will also apply to all other pigs in the herd and will vary from one APP to another (Hp being more sensitive to these effects than the other APPs studied, see above) [16,17,20,21]. Thus, although the present study as well as that of other investigators for example Parra et al. [18] provide values for normal, pre-infection concentrations of a number of useful APPs it is recommended that group/herd-specific data are always obtained and used in order to define cut-off values. Such data may be derived by continuous APP surveillance of herds in periods in which the herd is free from disease.

In addition, to further corroborate the conclusions on which APP combinations are generally optimal, future studies should extend to more, relevant infections including different types of (clinical and subclinical) viral infections, infections with helminths and bacterial infections restricted to the mucosal surfaces. This would generate a more complete picture of the possibilities and limitations of the use of APPs for revealing infection and to possibly define APP subsets that are particularly applicable to certain groups of infections and/or situations.

The potential of the method for analyzing APP data from herds in which knowledge of infections is scarce opens up new ways of classifying/certifying pig herds with improved welfare. In addition this would allow continuous, general screening of herds for health problems which may be followed up by traditional serological methods if needed. In order to achieve the full potential of this approach, validated and robust APP assays and APP standards need to be generally available.

## Competing interests

The authors declare that they have no competing interests.

## Authors' contributions

PH conceived, coordinated and designed the study, planned analyses and drafted the manuscript, AS carried out the statistical treatment of data and drafted the corresponding parts of the manuscript as well as the supplementary data (Additional file 1), MP, RC, FL, FC, PDE, MT, EG and NSS did immunoassays of acute phase proteins. In addition NSS collected and treated data, planned and coordinated the study and co-drafted the manuscript. All authors read and approved the final manuscript.

## Supplementary Material

Additional file 1 **Supplementary data**. All APP concentration data for all treatment groups. All detection probabilities for all treatment groups and for all APPs and their combinations. Performance index for APPs and APP combinations. Click here for file
