# Supplementary material for: Optimal combinations of acute phase proteins for detecting infectious disease in pigs
Source: Vet Res. 2011 Mar 17;42(1):50. doi: 10.1186/1297-9716-42-50 (PMC3072945; doi:10.1186/1297-9716-42-50)
Supplement: Additional file 1 — Supplementary data. All APP concentration data for all treatment groups. All detection probabilities for all treatment groups and for all APPs and their combinations. Performance index for APPs and APP combinations. [file 1297-9716-42-50-S1.DOC]

**SUPPLEMENTARY DATA**

A detailed description of the statistical approach can be obtained from the authors.

Table S1: Detection probabilities, all groups, all time points for the four selected APPs, single and combined.

**Actinobacillus pleuropneumoniae**

| protein | cut-off value *c* | day 0 | day 3 | day 7 | day 10 | day 14 | day 18 |
| --- | --- | --- | --- | --- | --- | --- | --- |
| CRP | 23.70 | 0.05 | 0.49 | 0.77 | 0.59 | 0.00 | 0.38 |
| Hp | 4.21 | 0.05 | 0.94 | 0.85 | 0.84 | 0.70 | 0.62 |
| -apoA1 | -1.84 | 0.05 | 0.99 | 0.33 | 0.16 | 0.43 | 0.01 |
| pigMAP | 0.45 | 0.05 | 0.99 | 0.99 | 0.96 | 0.96 | 0.98 |

| protein | dimension | cut-off | day 0 | day 3 | day 7 | day 10 | day 14 | day 18 |
| --- | --- | --- | --- | --- | --- | --- | --- | --- |
| CRP | 1 | 23.70 | 0.05 | 0.49 | 0.77 | 0.59 | 0.00 | 0.38 |
| Hp | 1 | 4.21 | 0.05 | 0.94 | 0.85 | 0.84 | 0.70 | 0.62 |
| -apoA1 | 1 | -1.84 | 0.05 | 0.99 | 0.33 | 0.16 | 0.43 | 0.01 |
| pigMAP | 1 | 0.45 | 0.05 | 0.99 | 0.99 | 0.96 | 0.96 | 0.98 |
| CRP,Hp | 2 | - | 0.05 | 0.97 | 0.94 | 0.89 | 0.70 | 0.76 |
| CRP,apoA1 | 2 | - | 0.05 | 0.99 | 0.79 | 0.61 | 0.38 | 0.29 |
| CRP,pigMAP | 2 | - | 0.05 | 0.99 | 0.99 | 0.98 | 0.96 | 0.99 |
| Hp,apoA1 | 2 | - | 0.05 | 1.00 | 1.00 | 1.00 | 1.00 | 1.00 |
| Hp,pigMAP | 2 | - | 0.05 | 1.00 | 1.00 | 1.00 | 1.00 | 1.00 |
| apoA1,pigMAP | 2 | - | 0.05 | 1.00 | 0.99 | 0.96 | 0.98 | 0.97 |
| CRP,Hp,apoA1 | 3 | - | 0.05 | 1.00 | 1.00 | 1.00 | 1.00 | 1.00 |
| CRP,Hp,pigMAP | 3 | - | 0.05 | 0.97 | 0.94 | 0.88 | 0.70 | 0.74 |
| CRP,apoA1,pigMAP | 3 | - | 0.05 | 1.00 | 0.99 | 1.00 | 1.00 | 1.00 |
| Hp,apoA1,pigMAP | 3 | - | 0.05 | 1.00 | 1.00 | 1.00 | 1.00 | 1.00 |
| CRP,Hp,apoA1,pigMAP | 4 | - | 0.05 | 1.00 | 1.00 | 1.00 | 1.00 | 1.00 |

**Mycoplasma hyosynoviae**

| protein | cut-off value *c* | day -16 | day -2 | day 4 | day 6 | day 7 | day 9 | day 12 | day 14 | day 15 |
| --- | --- | --- | --- | --- | --- | --- | --- | --- | --- | --- |
| CRP | 0.55 | 0.05 | 0.34 | 0.68 | 0.78 | 0.70 | 0.84 | 0.65 | 0.67 | 0.75 |
| Hp | 7.42 | 0.05 | 0.76 | 0.00 | 0.00 | 0.72 | 0.93 | 0.76 | 0.69 | 0.00 |
| -apoA1 | -2.92 | 0.05 | 0.65 | 0.97 | 0.72 | 1.00 | 0.99 | 0.97 | 0.81 | 0.37 |
| pigMAP | 0.82 | 0.05 | 0.15 | 0.25 | 0.02 | 0.34 | 0.46 | 0.70 | 0.36 | 0.40 |

| protein | dimension | cut-off | day -16 | day -2 | day 4 | day 6 | day 7 | day 9 | day 12 | day 14 | day 15 |
| --- | --- | --- | --- | --- | --- | --- | --- | --- | --- | --- | --- |
| CRP | 1 | 0.55 | 0.05 | 0.34 | 0.68 | 0.78 | 0.70 | 0.84 | 0.65 | 0.67 | 0.75 |
| Hp | 1 | 4.21 | 0.05 | 0.76 | 0.00 | 0.00 | 0.72 | 0.93 | 0.76 | 0.69 | 0.00 |
| -apoA1 | 1 | -1.84 | 0.05 | 0.65 | 0.97 | 0.72 | 1.00 | 0.99 | 0.97 | 0.81 | 0.37 |
| pigMAP | 1 | 0.45 | 0.05 | 0.15 | 0.25 | 0.02 | 0.34 | 0.46 | 0.70 | 0.36 | 0.40 |
| CRP,Hp | 2 | - | 0.05 | 0.83 | 0.69 | 0.77 | 0.94 | 0.98 | 0.97 | 0.97 | 1.00 |
| CRP,apoA1 | 2 | - | 0.05 | 0.56 | 0.71 | 0.81 | 0.73 | 0.84 | 0.70 | 0.66 | 0.51 |
| CRP,pigMAP | 2 | - | 0.05 | 0.48 | 0.98 | 0.98 | 0.98 | 1.00 | 0.99 | 0.97 | 1.00 |
| Hp,apoA1 | 2 | - | 0.05 | 0.76 | 0.90 | 0.67 | 0.71 | 0.93 | 0.77 | 0.70 | 1.00 |
| Hp,pigMAP | 2 | - | 0.05 | 1.00 | 0.22 | 0.01 | 1.00 | 1.00 | 1.00 | 1.00 | 1.00 |
| apoA1,pigMAP | 2 | - | 0.05 | 0.63 | 0.94 | 0.66 | 1.00 | 0.99 | 0.94 | 0.82 | 1.00 |
| CRP,Hp,apoA1 | 3 | - | 0.05 | 0.82 | 0.75 | 0.80 | 0.94 | 0.97 | 0.97 | 0.97 | 0.97 |
| CRP,Hp,pigMAP | 3 | - | 0.05 | 1.00 | 0.97 | 0.96 | 1.00 | 1.00 | 1.00 | 1.00 | 1.00 |
| CRP,apoA1,pigMAP | 3 | - | 0.05 | 0.66 | 0.93 | 0.98 | 1.00 | 1.00 | 1.00 | 1.00 | 1.00 |
| Hp,apoA1,pigMAP | 3 | - | 0.05 | 0.77 | 0.88 | 0.62 | 0.72 | 0.93 | 0.77 | 0.74 | 0.82 |
| CRP,Hp,apoA1,pigMAP | 4 | - | 0.05 | 0.87 | 0.99 | 1.00 | 0.98 | 0.99 | 0.98 | 1.00 | 1.00 |

**Streptococcus suis**

| protein | cut-off value *c* | day -8 | day 0 | day 1 | day 2 | day 5 | day 11 | day 12 | day 14 |
| --- | --- | --- | --- | --- | --- | --- | --- | --- | --- |
| CRP | 30.82 | 0.05 | 0.00 | 0.87 | 0.89 | 0.89 | 0.77 | 0.57 | 0.44 |
| Hp | 941.49 | 0.05 | 0.11 | 0.85 | 0.85 | 0.99 | 0.99 | 0.87 | 0.95 |
| -AP0A1 | -0.96 | 0.05 | 0.00 | 0.17 | 0.46 | 0.05 | 0.01 | 0.05 | 0.00 |
| pigMAP | 1.55 | 0.05 | 0.00 | 0.93 | 0.99 | 0.03 | 0.97 | 0.77 | 0.60 |

| protein | dimension | cut-off | day -8 | day 0 | day 1 | day 2 | day 5 | day 8 | day 12 | day 14 |
| --- | --- | --- | --- | --- | --- | --- | --- | --- | --- | --- |
| CRP | 1 | 30.82 | 0.05 | 0.00 | 0.87 | 0.89 | 0.89 | 0.77 | 0.57 | 0.44 |
| Hp | 1 | 941.49 | 0.05 | 0.11 | 0.85 | 0.85 | 0.99 | 0.99 | 0.87 | 0.95 |
| -apoA1 | 1 | -0.96 | 0.05 | 0.00 | 0.17 | 0.46 | 0.05 | 0.01 | 0.05 | 0.00 |
| pigMAP | 1 | 1.55 | 0.05 | 0.00 | 0.93 | 0.99 | 0.03 | 0.97 | 0.77 | 0.60 |
| CRP,Hp | 2 | - | 0.05 | 0.11 | 0.94 | 0.99 | 1.00 | 1.00 | 0.93 | 0.98 |
| CRP,apoA1 | 2 | - | 0.05 | 0.00 | 1.00 | 0.99 | 0.94 | 0.82 | 0.67 | 0.49 |
| CRP,pigMAP | 2 | - | 0.05 | 0.00 | 1.00 | 1.00 | 0.99 | 0.99 | 0.90 | 0.78 |
| Hp,apoA1 | 2 | - | 0.05 | 0.09 | 0.95 | 0.99 | 1.00 | 1.00 | 0.86 | 0.93 |
| Hp,pigMAP | 2 | - | 0.05 | 0.09 | 1.00 | 1.00 | 1.00 | 1.00 | 0.96 | 0.94 |
| apoA1,pigMAP | 2 | - | 0.05 | 0.00 | 0.98 | 1.00 | 0.99 | 0.98 | 0.83 | 0.65 |
| CRP,Hp,apoA1 | 3 | - | 0.05 | 0.08 | 1.00 | 1.00 | 1.00 | 1.00 | 0.92 | 0.98 |
| CRP,Hp,pigMAP | 3 | - | 0.05 | 0.08 | 0.98 | 1.00 | 1.00 | 1.00 | 0.98 | 0.98 |
| CRP,apoA1,pigMAP | 3 | - | 0.05 | 0.00 | 1.00 | 1.00 | 0.99 | 0.99 | 0.91 | 0.76 |
| Hp,apoA1,pigMAP | 3 | - | 0.05 | 0.08 | 1.00 | 1.00 | 1.00 | 1.00 | 0.99 | 0.95 |
| CRP,Hp,apoA1,pigMAP | 4 | - | 0.05 | 0.07 | 1.00 | 1.00 | 1.00 | 1.00 | 0.99 | 0.98 |

**Toxoplasma gondii**

| protein | cut-off value *c* | day -4 | day 3 | day 6 | day 8 | day 10 | day 14 | day 17 | day 21 |
| --- | --- | --- | --- | --- | --- | --- | --- | --- | --- |
| CRP | 14.41 | 0.05 | 0.00 | 0.68 | 0.89 | 0.95 | 0.00 | 0.02 | 0.00 |
| Hp | 1541.43 | 0.05 | 0.09 | 0.52 | 0.90 | 0.70 | 0.50 | 0.24 | 0.00 |
| -apoA1 | -2.06 | 0.05 | 0.00 | 0.17 | 0.94 | 0.13 | 0.01 | 0.00 | 0.01 |
| pigMAP | 0.59 | 0.05 | 0.00 | 0.69 | 1.00 | 0.99 | 0.94 | 0.62 | 0.14 |

| protein | dimension | cut-off | day -4 | day 3 | day 6 | day 8 | day 10 | day 14 | day 17 | day 21 |
| --- | --- | --- | --- | --- | --- | --- | --- | --- | --- | --- |
| CRP | 1 | 14.24 | 0.05 | 0.00 | 0.68 | 0.90 | 0.95 | 0.00 | 0.02 | 0.00 |
| Hp | 1 | 1524.90 | 0.05 | 0.09 | 0.52 | 0.90 | 0.70 | 0.50 | 0.24 | 0.00 |
| -apoA1 | 1 | -0.59 | 0.05 | 0.00 | 0.18 | 0.95 | 0.13 | 0.01 | 0.00 | 0.01 |
| pigMAP | 1 | 2.07 | 0.05 | 0.00 | 0.69 | 1.00 | 0.99 | 0.94 | 0.62 | 0.15 |
| CRP,Hp | 2 | - | 0.05 | 0.07 | 0.87 | 1.00 | 0.97 | 0.50 | 0.21 | 0.00 |
| CRP,apoA1 | 2 | - | 0.05 | 0.00 | 0.67 | 0.89 | 0.94 | 0.06 | 0.02 | 0.04 |
| CRP,pigMAP | 2 | - | 0.05 | 0.00 | 0.77 | 1.00 | 0.99 | 0.94 | 0.61 | 0.14 |
| Hp,apoA1 | 2 | - | 0.05 | 0.08 | 0.53 | 1.00 | 0.82 | 0.62 | 0.22 | 0.01 |
| Hp,pigMAP | 2 | - | 0.05 | 0.08 | 0.91 | 1.00 | 1.00 | 1.00 | 0.85 | 0.21 |
| apoA1,pigMAP | 2 | - | 0.05 | 0.00 | 0.78 | 1.00 | 0.99 | 0.94 | 0.68 | 0.23 |
| CRP,Hp,apoA1 | 3 | - | 0.05 | 0.08 | 0.86 | 1.00 | 0.97 | 0.86 | 0.19 | 0.08 |
| CRP,Hp,pigMAP | 3 | - | 0.05 | 0.05 | 0.92 | 1.00 | 1.00 | 1.00 | 0.86 | 0.26 |
| CRP,apoA1,pigMAP | 3 | - | 0.05 | 0.00 | 0.98 | 1.00 | 1.00 | 0.97 | 0.73 | 0.33 |
| Hp,apoA1,pigMAP | 3 | - | 0.05 | 0.07 | 0.94 | 1.00 | 1.00 | 1.00 | 0.95 | 0.32 |
| CRP,Hp,apoA1,pigMAP | 4 | - | 0.05 | 0.04 | 0.98 | 1.00 | 1.00 | 1.00 | 0.95 | 0.50 |

**Inflammation**

| protein | cut-off value *c* | day 0 | day 0.5 | day 1 | day 1.5 | day 2 | day 3 | day 4 | day 7 | day 10 | day 14 |
| --- | --- | --- | --- | --- | --- | --- | --- | --- | --- | --- | --- |
| CRP | 19.39 | 0.05 | 0.60 | 1.00 | 1.00 | 1.00 | 1.00 | 0.98 | 0.37 | 0.17 | 0.30 |
| Hp | 853.02 | 0.05 | 0.04 | 0.75 | 0.95 | 0.96 | 0.94 | 0.88 | 0.53 | 0.04 | 0.28 |
| -apoA1 | -1.62 | 0.05 | 0.07 | 0.29 | 0.42 | 0.30 | 0.15 | 0.06 | 0.01 | 0.02 | 0.00 |
| pigMAP | 0.93 | 0.05 | 0.06 | 0.73 | 0.92 | 0.96 | 0.96 | 0.94 | 0.90 | 0.61 | 0.13 |

| protein | dimension | cut-off | day 0 | day 0.5 | day 1 | day 1.5 | day 2 | day 3 | day 4 | day 7 | day 10 | day 14 |
| --- | --- | --- | --- | --- | --- | --- | --- | --- | --- | --- | --- | --- |
| CRP | 1 | 19.39 | 0.05 | 0.60 | 1.00 | 1.00 | 1.00 | 1.00 | 0.98 | 0.37 | 0.17 | 0.30 |
| Hp | 1 | 853.02 | 0.05 | 0.04 | 0.75 | 0.95 | 0.96 | 0.94 | 0.88 | 0.53 | 0.04 | 0.28 |
| -apoA1 | 1 | -0.93 | 0.05 | 0.07 | 0.29 | 0.42 | 0.30 | 0.15 | 0.06 | 0.01 | 0.02 | 0.00 |
| pigMAP | 1 | 1.62 | 0.05 | 0.06 | 0.73 | 0.92 | 0.96 | 0.96 | 0.94 | 0.90 | 0.61 | 0.13 |
| CRP,Hp | 2 | - | 0.05 | 0.57 | 1.00 | 1.00 | 1.00 | 1.00 | 0.99 | 0.70 | 0.16 | 0.40 |
| CRP,apoA1 | 2 | - | 0.05 | 0.56 | 1.00 | 1.00 | 1.00 | 1.00 | 0.98 | 0.33 | 0.14 | 0.26 |
| CRP,pigMAP | 2 | - | 0.05 | 0.55 | 1.00 | 1.00 | 1.00 | 1.00 | 1.00 | 0.90 | 0.58 | 0.25 |
| Hp,apoA1 | 2 | - | 0.05 | 0.10 | 0.78 | 0.96 | 0.99 | 0.99 | 0.95 | 0.65 | 0.05 | 0.30 |
| Hp,pigMAP | 2 | - | 0.05 | 0.12 | 0.80 | 0.97 | 0.98 | 0.97 | 0.96 | 0.85 | 0.72 | 0.32 |
| apoA1,pigMAP | 2 | - | 0.05 | 0.09 | 0.74 | 0.90 | 0.95 | 0.95 | 0.94 | 0.89 | 0.69 | 0.20 |
| CRP,Hp,apoA1 | 3 | - | 0.05 | 0.52 | 1.00 | 1.00 | 1.00 | 1.00 | 0.99 | 0.83 | 0.26 | 0.52 |
| CRP,Hp,pigMAP | 3 | - | 0.05 | 0.58 | 1.00 | 1.00 | 1.00 | 1.00 | 1.00 | 0.90 | 0.69 | 0.36 |
| CRP,apoA1,pigMAP | 3 | - | 0.05 | 0.55 | 1.00 | 1.00 | 1.00 | 1.00 | 0.99 | 0.94 | 0.80 | 0.50 |
| Hp,apoA1,pigMAP | 3 | - | 0.05 | 0.14 | 0.77 | 0.97 | 0.99 | 0.99 | 0.98 | 0.92 | 0.75 | 0.44 |
| CRP,Hp,apoA1,pigMAP | 4 | - | 0.05 | 0.56 | 1.00 | 1.00 | 1.00 | 1.00 | 1.00 | 1.00 | 0.99 | 0.98 |

Table S2: Performance index for all possible APP combinations.

| APP combination 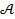 | Performance index 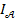 |
| --- | --- |
| CRP | 0.52 |
| Hp | 0.57 |
| apoA1 | 0.30 |
| pigMAP | 0.63 |
| CRP, Hp | 0.72 |
| CRP, apoA1 | 0.57 |
| CRP, pigMAP | 0.81 |
| Hp, apoA1 | 0.70 |
| Hp, pigMAP | 0.77 |
| apoA1, pigMAP | 0.78 |
| CRP, Hp, apoA1 | 0.77 |
| CRP, Hp, pigMAP | 0.82 |
| CRP, apoA1 ,pigMAP | 0.84 |
| Hp, apoA1, pigMAP | 0.81 |
| CRP, Hp, apoA1,pigMAP | 0.89 |

Figure S1: Raw data for individual animals, all treatments groups for the four selected APPs.

Figure S2: Integral of detection probability function
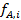
, with
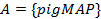
,
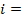
Inflammation, and
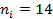
.

Figure S3a: Detection probabilities for the four APPs, for different infection types.

Figure S3b: Detection probabilities for the optimal APP combinations of size 1, 2, 3 and 4, for the different infection agents and inflammation.

Figure S4: All APP combinations, detection probabilities in the different treatment groups.

**Four APP combination**

**Three APP combinations**

**Two APP combinations**
